# Exploring Participants’ Experiences of Digital Health Interventions With Qualitative Methods: Guidance for Researchers

**DOI:** 10.2196/62761

**Published:** 2024-11-28

**Authors:** Kristin Harrison Ginsberg, Katie Babbott, Anna Serlachius

**Affiliations:** 1 Department of Psychological Medicine University of Auckland Auckland New Zealand

**Keywords:** qualitative methods, content analysis, thematic analysis, digital health evaluation, user engagement, user experience, digital health intervention, innovation, patient experience, health care, researcher, technology, mobile health, mHealth, telemedicine, digital health, behavior change, usability, tutorial, research methods, qualitative research, study design

## Abstract

Digital health interventions have gained prominence in recent years, offering innovative solutions to improve health care delivery and patient outcomes. Researchers are increasingly using qualitative approaches to explore patient experiences of using digital health interventions. Yet, the qualitative methods used in these studies can vary widely, and some methods are frequently misapplied. We highlight the methods we find most fit for purpose to explore user experiences of digital tools and propose 5 questions for researchers to use to help them select a qualitative method that best suits their research aims.

## Introduction

Digital health interventions encompass a wide range of technologies, such as mobile apps, websites, wearable devices, and telemedicine platforms. Their use in behavioral change and mental health interventions are common; such interventions are increasingly used as both an adjunct to face-to-face care and as standalone interventions [1-4]. Although digital health interventions have ostensible benefits in terms of scalability and potential to improve equity of access, the high dropout rates of digital health interventions, which can reach up to 80% [5,6], raise questions of acceptability and usability. Understanding participants’ experiences in these interventions is therefore crucial for developing effective digital tools, improving and tailoring existing digital tools, and optimizing health outcomes.

Qualitative research exploring user experience of digital health tools has surged alongside the exponential increase in digital health interventions. Researchers frequently use qualitative approaches to explore engagement, usability, and uptake of digital health interventions. In this type of “applied research,” researchers often start with predefined research questions, such as how well an intervention works and under what circumstances [7]. Yet, despite these focused research questions, the qualitative methods (ie, the qualitative approaches used) and methodology (ie, the theoretical rationale and perspective that guide the research) used in these studies can vary widely and be misapplied. For example, a systematic review of 16 studies that used qualitative methods to assess user experiences of digital interventions for pediatric patients [8] found an eclectic range of data analysis methods used, including thematic analysis (37%), content analysis (31%), hermeneutic research analysis (6%), and the generically described “deductive” analysis (6%). Nearly 20% of the articles did not describe their qualitative analysis methodology (ie, their theoretically informed approach). These vague, incomplete, or absent descriptions of qualitative methodology are common across the health intervention literature [9].

As health psychology researchers who have conducted a range of studies exploring patient experiences using digital health interventions [10,11], we understand the challenge of selecting a qualitative approach for this type of research, as there are no existing guidelines to inform the selection of the right qualitative method (ie, the approach that is used) and/or methodology (ie, the overarching philosophical framework or lens). Although there exist several guidelines for improving the reporting of qualitative research [12-15], these generally do not offer guidance on selecting appropriate qualitative approaches. Furthermore, qualitative approaches are also more flexible and interpretative than quantitative methods, which can add to selection confusion. Therefore, in this article, we summarize the most common qualitative approaches used in digital health research when exploring user engagement and make recommendations based on our experiences. We also provide 5 questions and a decision tree that digital health intervention researchers can use to select a qualitative approach that best meets their research goals.

## First Things First: Grounded in Theory, or Not?

As qualitative research has grown in popularity in behavioral research, so too has confusion over the range of approaches available and the theoretical or methodological frameworks involved. For example, frequent misapplication of thematic analysis, one of the most often used qualitative methods, has led Braun and Clarke [16], authors of the seminal paper on the method, to publish 4 recent papers clarifying its methodology and “flexible” theoretical approach [17-20].

Starting first with theory and its underlying epistemology (ie, underlying philosophy) can help you choose the qualitative approach that best fits your research question. Qualitative researchers exploring participant experiences in health-related research often take either a constructionist or realist approach. A constructionist approach, also referred to as interpretivist, critical, or “artfully interpretive” [21], assumes that reality is socially constructed and shaped through interactions, language, and meaning-making processes. It emphasizes that individuals and groups actively create their own realities through their interpretations and interactions with the world. Social constructionist qualitative approaches in health- and mental health–related research often analyze patterns in language, discourse, and narratives to explore the underlying meaning and the social constructs of reality [22].

In contrast, a realist approach, or a “scientifically descriptive” [21] approach, posits that there is a degree of objectivity that exists independently of human perception. It suggests that social phenomena have inherent structures and properties that exist regardless of human interpretation. Realist approaches are often used in mixed methods research and tend to analyze qualitative data with a focus on identifying a “consistency of meaning,” often through triangulation and the use of several co-coders [23].

Researchers also need to consider whether they are taking a deductive (top-down), inductive (data-driven), or abductive (combination of both) approach in their analysis and whether they will focus on semantic (explicit) or latent (implicit) meanings in the data. It is increasingly acknowledged that the coding and analysis process is rarely completely inductive or deductive [24] and is usually a combination of both [25,26]. For example, even if you choose a mainly deductive approach, it is not uncommon that unexpected and largely inductive (data-driven) codes may also become apparent during the qualitative coding process, even if you are coding with a framework in mind or are approaching the data with predetermined deductive codes. This “abductive” approach also allows for a more nuanced and contextual story to be told. For example, even if you are coding participants’ responses using a digital usability framework, you can also code inductively when participants raise important concepts, experiences, and thoughts that relate to the overall research question and aims.

## Untangling Qualitative Methodologies

### Overview

Some qualitative approaches are inherently linked to their broader methodological frameworks (eg, narrative analysis, interpretative phenomenological analysis, and grounded theory) while others are more flexible in terms of belonging to a certain methodological framework (eg, thematic analysis [16] and content analysis [27]). Thematic analysis, qualitative content analysis, grounded theory, and interpretative phenomenological analysis are some of the many forms of pattern-seeking qualitative approaches. In our opinion, grounded theory and interpretative phenomenological analysis, which are highly interpretive and explorative, are less likely to fit the needs of most digital health research exploring participant usability of and engagement with digital tools, and we are not going to discuss them here. (Good guidance on these methods can be found via Strauss and Corbin [28] and McLeod [29].) Instead, we’ve highlighted the 2 most commonly used approaches in digital health research exploring participant experience and user engagement.

### Qualitative Content Analysis

Qualitative content analysis, including conventional, directed, and summative approaches [30], uses systematic methods to analyze patterns in text and explore meaning. Sometimes considered an “intermediary” approach between qualitative and quantitative methods [31], content analysis, particularly some types, such as summative, use methods that borrow from quantitative research, such as counting the frequency of words or phrases [30]. Researchers using content analysis may take a deductive, inductive, or abductive (combined) approach and often use it deductively given its utility for exploring predefined categories and/or incorporating an existing behavioral or theoretical framework [27,29]. Conventional content analysis can also be used more interpretively, and shares some similarities with certain types of thematic analysis (such as the framework method, discussed below) [32].

We find qualitative content analysis particularly useful in digital health intervention evaluations that build on existing frameworks or previous research (as most of our work does). We have also found directed content analysis to be an efficient and effective method to analyze qualitative data within mixed methods randomized controlled trials of digital health interventions (such as in Brenton-Peters et al [33] and Serlachius et al [34]).

### Thematic Analysis

Similar in some aspects to content analysis, thematic analysis is a “family” of related methods [19] that involve labeling (“coding”) data and then organizing them into themes (patterns of meaning). Reflexive thematic analysis, which directly addresses the researcher’s role and process in the analysis, is Braun and Clarke’s [16] updated approach and seeks in part to differentiate itself from older styles of thematic analysis, such as codebook and framework analysis, which take a more structured, and often combined inductive/deductive, approach.

Due in part to Braun and Clarke’s [16] detailed, step-by-step description of thematic analysis published in 2006, this approach has become widely used. They have written at length on how this approach is frequently misunderstood and misapplied, as demonstrated by a recent study where they reviewed 20 health-related studies that used thematic analysis and found the most common problem was in the creation of themes [20]. In reflexive thematic analysis, a theme should highlight a broader meaning across the dataset, telling an interpretive story. However, many studies confuse themes with “topic summaries,” such as “helpfulness of the intervention” or “ease of use.” Braun and Clarke [20] note that if the theme could have been created before conducting the data analysis (ie, it could be mapped directly to an interview question), then it is a topic summary.

We feel that if your research question and aims align best with topic summaries and deductive analysis, qualitative content analysis or codebook or framework thematic analysis may be a better fit than reflexive thematic analysis or other more interpretative approaches.

## Selecting a Qualitative Approach: 5 Questions to Consider

**Figure 1:**
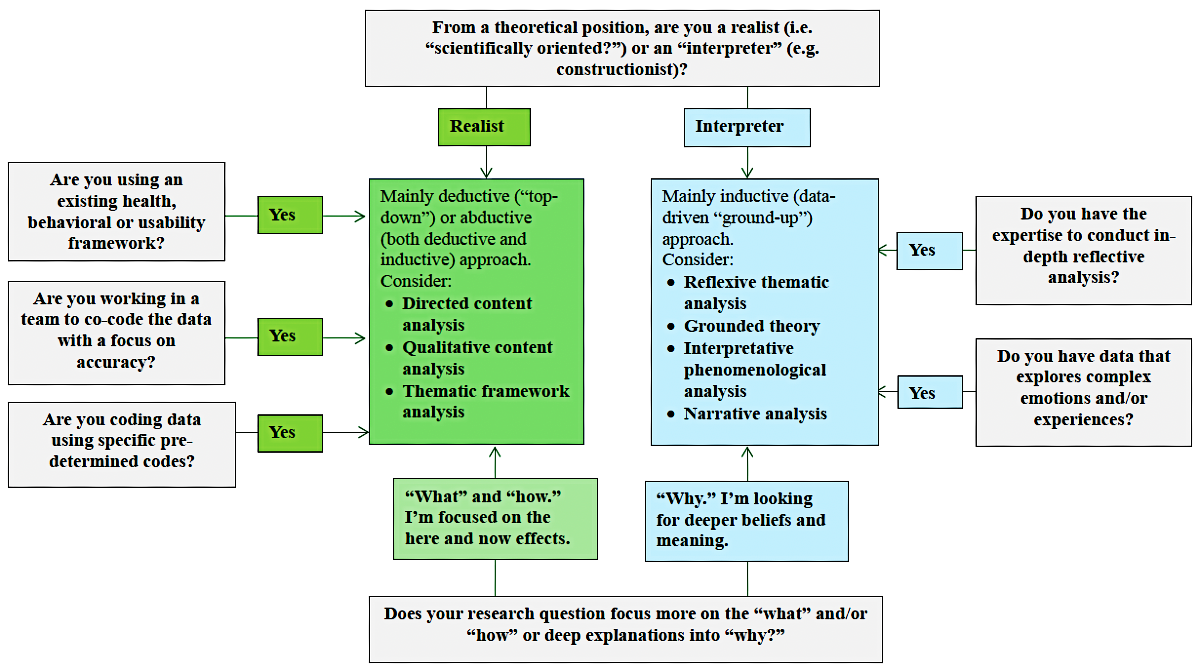
Questions to help select a qualitative method in digital health research evaluation.

To use qualitative approaches more effectively in digital health user engagement research, we believe there needs to be more coherence and consistency in choosing qualitative methods. Therefore, we suggest researchers use the following 5 questions to help select the qualitative approach most appropriate for their research aims (Figure 1 shows a decision tree.)

### Question 1: What Is Your Theoretical/Methodological Position: Are You a Realist or an Interpreter?

Most digital health intervention evaluation studies have pragmatic goals grounded in a realist (ie, “scientifically oriented”) view, building on previous research findings, for example, as part of mixed methods research. If your study fits within this orientation, strongly consider using a method like content analysis or thematic framework analysis, which can flexibly accommodate existing frameworks and build on previous quantitative findings.

While more inductively aligned methods of thematic analysis or other more interpretative approaches such as narrative analysis or grounded theory can be excellent approaches at the development stage of a digital health intervention, when there is more emphasis on exploring in-depth patient experiences, they are frequently less aligned with most research aims around digital health intervention evaluation and exploring questions regarding user experience and user engagement through a realist epistemology. However, there are exceptions; for example, a study by Knox et al [35] explored stakeholder views on virtual pulmonary rehabilitation using a critical epistemology, and a paper by Bleyel et al [36] explored patient perceptions of mental health care through video consultations from a critical realist perspective [36]. These examples demonstrate the importance of articulating your research aims, methods, and methodological perspective to justify your chosen approach, which is not always easily achievable within the tight word-count limits of medical journals.

Also carefully consider whether you want to take a deductive, inductive, or abductive approach in your analysis. While you can use any of these approaches across the different types of content and thematic analysis methods [27,37], you should select an analysis method that works within your research aims and state this orientation in your reported methodology.

### Question 2: Are You Using an Existing Health or Behavioral Change Framework?

Many digital health interventions are built on existing intervention or behavior change frameworks, such as the capability, opportunity, motivation-behavior (COM-B) model [38] and the reach, effectiveness, adoption, implementation, and maintenance (RE-AIM) framework [39]. If you want to use an existing framework in your qualitative analysis, as many digital intervention studies do, choose an approach designed for this, such as directed content analysis or thematic framework analysis. Do not choose a highly interpretive approach, such as reflexive thematic analysis or interpretative phenomenological analysis, if you intend to map your qualitative findings onto an existing framework. A good example by Szinay and colleagues [40] used the framework method to explore engagement with well-being apps informed by the COM-B model. For further details regarding the framework method, refer to Gale and colleagues [32], who provide a comprehensive outline of the 7 stages of analysis using this method in health research.

### Question 3: What Type of Research Question Are You Trying to Answer?

Qualitative research is well suited to answer “how” and “why” questions, such as why individuals did (or did not) use a digital health intervention and how they went about it. But different research questions may be better suited to one type of qualitative analysis than another.

For example, let us suppose your digital health intervention for weight loss had a high drop-out rate, and you want to use qualitative methods to explore reasons why. The most suitable qualitative approach depends on your underlying rationale and research strategy (including your methodological/theoretical position) and what you plan to do in response to your findings (ie, changes or iterations to the intervention or exploring a different approach altogether). If you want to explore topics such as participants’ beliefs about weight loss and/or perceived barriers to weight loss, narrative analysis or another more inductive approach may be more appropriate. However, if, as is more common in digital health intervention evaluation research, you have specific questions related to participants’ opinions about the design, content, and activities within the intervention itself that caused them to stop using it, then a more focused and deductive or abductive qualitative approach (ie, content analysis) may be more useful.

### Question 4: How Are You Collecting Data?

According to a 2019 systematic review, frequently used qualitative data collection tools in digital health intervention evaluation and testing and exploring users’ perspectives include “think-aloud” protocols, focus groups, and interviews [41]. We often also use open-ended online survey questions in our mixed methods evaluation studies. It is important to consider the type of data you are collecting alongside your analysis plan.

Stemming from cognitive psychology, the think-aloud method asks participants to share what they are thinking while performing a task [42], which can lead to a focused data set. Because of this, the think-aloud method is popular in usability research and has been found to successfully generate intervention improvements [43]. Think-aloud data are most commonly analyzed using a deductive approach and with methods similar to content analysis, such as counting positive or negative sentiment [44], but these data can also be coded and organized into topics or themes [12].

Focus groups and interviews can generate large datasets that may cover a broad range of topics and experiences. While exploratory questions may be useful in digital health intervention development research, a more focused approach is usually necessary to meet the goals of evaluation research or exploring whether a digital tool met participants’ needs. Therefore, it is critical to develop an interview guide that considers your research questions, theoretical framework, and desired result type (ie, themes or frequencies). Otherwise, you may end up with a wide-ranging and meandering dataset that does not answer your research questions and leads to what Braun and Clarke [20] call a “mish-mash” of ineffective qualitative analysis.

### Question 5: How Much Time Do You Have?

Digital health interventions often require rapid evolution to stay relevant, and researchers may have limited time, resources, and funding [7]. Therefore, it is important to consider how quickly you need to generate your results, as well as the resources needed to complete the analysis.

In terms of the approaches discussed here, reflexive thematic analysis, interpretive phenomenological analysis, grounded theory, and narrative analysis often require significant time to conduct, with multiple rounds of detailed, in-depth coding and thematic review and ongoing exploration regarding reflexivity and how it influences the process and outcomes of the analysis. Content analysis or framework thematic analysis can be done in a more efficient and timely manner, with codebooks often developed in advance of analysis and the frequent use of co-coders with a focus on accuracy.

Increasingly, so-called rapid approaches have been developed and compared against more traditional qualitative approaches due to this need to reduce time and improve efficiency in health care research [45,46]. For example, Holdsworth et al [47] used a rapid form of framework analysis to summarize evaluation data from intensive care unit site visits while still on site. They found this approach delivered significant savings in analysis and transcription costs.

## Conclusion

After more than a decade of conducting qualitative research exploring patient experiences of and user engagement with digital health tools, we too have grappled with choosing appropriate qualitative methods. In this article, we draw on our experience, which has been largely in health psychology and digital health–related research, but acknowledge that similar qualitative design challenges exist in health informatics, health services research, and the broader usability research literature [14,15]. Ultimately, we believe that many of these ongoing interdisciplinary challenges and discussions enhance overall understanding of the benefits of qualitative and mixed methods research and lead to improvements not just in the conducting and reporting of qualitative research but also in the ongoing development and clarification of the methods themselves. This paper did not have the scope to explore questions regarding what constitutes “good” or rigorous qualitative research in digital health, but there are several articles providing useful summaries of key criteria in digital health research, health services research, and digital health assessment [48,49].

Digital health interventions offer innovative solutions to improve health care delivery and patient outcomes. Researchers now frequently use qualitative approaches to explore engagement, usability, and uptake of digital health interventions. However, some of the most popular qualitative approaches are frequently misapplied in this type of research and may not be the most appropriate for this type of research. Therefore, we suggest researchers consider their research aims, theoretical orientation, and the time and resources available before selecting a qualitative approach to use when exploring participants’ experiences of digital tools.

## Abbreviations

COM-B: capability, opportunity, motivation-behavior
Re-Aim: reach, effectiveness, adoption, implementation, and maintenance
